# Admission Lipoprotein-Associated Phospholipase A_2_ Activity Is Not Associated with Long-Term Clinical Outcomes after ST-Segment Elevation Myocardial Infarction

**DOI:** 10.1371/journal.pone.0096251

**Published:** 2014-05-01

**Authors:** Pier Woudstra, Peter Damman, Wichert J. Kuijt, Wouter J. Kikkert, Maik J. Grundeken, Peter M. van Brussel, An K. Stroobants, Jan P. van Straalen, Johan C. Fischer, Karel T. Koch, José P. S. Henriques, Jan J. Piek, Jan G. P. Tijssen, Robbert J. de Winter

**Affiliations:** 1 Heart Center, Academic Medical Center – University of Amsterdam, Amsterdam, The Netherlands; 2 Department of Clinical Chemistry, Academic Medical Center – University of Amsterdam, Amsterdam, The Netherlands; University of Milan, Italy

## Abstract

**Background:**

Lipoprotein-associated phospholipase A_2_ (Lp-PLA_2_) activity is a biomarker predicting cardiovascular diseases in a real-world. However, the prognostic value in patients undergoing primary percutaneous coronary intervention (pPCI) for ST-segment elevation myocardial infarction (STEMI) on long-term clinical outcomes is unknown.

**Methods:**

Lp-PLA_2_ activity was measured in samples obtained prior to pPCI from consecutive STEMI patients in a high-volume intervention center from 2005 until 2007. Five years all-cause mortality was estimated with the Kaplan-Meier method and compared among tertiles of Lp-PLA2 activity during complete follow-up and with a landmark at 30 days. In a subpopulation clinical endpoints were assessed at three years. The prognostic value of Lp-PLA_2_, in addition to the Thrombolysis In Myocardial Infarction or multimarker risk score, was assessed in multivariable Cox regression.

**Results:**

The cohort (n = 987) was divided into tertiles (low <144, intermediate 144–179, and high >179 nmol/min/mL). Among the tertiles differences in baseline characteristics associated with long-term mortality were observed. However, no significant differences in five years mortality in association with Lp-PLA_2_ activity levels were found; intermediate versus low Lp-PLA_2_ (HR 0.97; CI 95% 0.68–1.40; p = 0.88) or high versus low Lp-PLA_2_ (HR 0.75; CI 95% 0.51–1.11; p = 0.15). Both in a landmark analysis and after adjustments for the established risk scores and selection of cases with biomarkers obtained, non-significant differences among the tertiles were observed. In the subpopulation no significant differences in clinical endpoints were observed among the tertiles.

**Conclusion:**

Lp-PLA_2_ activity levels at admission prior to pPCI in STEMI patients are not associated with the incidence of short and/or long-term clinical endpoints. Lp-PLA_2_ as an independent and clinically useful biomarker in the risk stratification of STEMI patients still remains to be proven.

## Introduction

Lipoprotein-associated phospholipase A_2_ (Lp-PLA_2_) is an enzyme secreted by monocyte-derived macrophages, T-lymphocytes, and mast cells, and bound mainly to LDL cholesterol, in particular small, dense LDL particles [1]. It is strongly expressed in the necrotic core and surrounding macrophages around vulnerable and ruptured atherosclerotic plaques [2]. Lp-PLA_2_ plays a major role in the pathophysiology of atherosclerosis, from initiation up to the development of cardiovascular complications [3]. In a meta-analysis of studies including patients with or without vascular disease, higher Lp-PLA_2_ mass or activity levels were linked to an increased mortality [4]. However, the clinical application of Lp-PLA_2_ mass or activity measurements remains subject of debate [5].

Within the spectrum of acute coronary syndrome (ACS), the prognosis of ST-segment elevation myocardial infarction (STEMI) patients (excluding shock cases) who are revascularized promptly with primary percutaneous coronary intervention (pPCI) is widely perceived as being good [6]. Over the years, the prognosis of STEMI patients has improved, although a significant proportion of these patients die before any medical contact [7]. However, patients at high risk for recurrent events remain challenging subgroups. The identification of these high risk subgroups could be helpful in further improvement of the prognosis of STEMI patients. A simple multimarker risk score based on estimated glomerular filtration rate (eGFR), glucose and N-terminal pro-brain natriuretic peptide (NTproBNP) could identify a subgroup of patients at high risk for mortality [8], [9]. These biomarkers respectively reflect renal function, glucose metabolism and left ventricular dysfunction. Among other known predictors of outcome in STEMI are several inflammatory markers such as interleukin-6 and −10 [10], and the controversial CRP [11], [12]. The Lp-PLA_2_ activity assay was made available to be the first to analyse the prognostic value of admission Lp-PLA_2_ on long-term clinical endpoints in patients presenting with STEMI treated with pPCI. Because of the existing data on the prognostic value of inflammatory markers and the knowledge of Lp-PLA_2_, we hypothesized that Lp-PLA_2_ activity can potentially contribute to the prognostic value of our multiple biomarker approach.

Hence, in the current analyses we investigate the independent prognostic value of Lp-PLA_2_ activity on long-term mortality in patients undergoing primary percutaneous coronary intervention (pPCI) for STEMI.

## Methods

### Source Population and Procedure Characteristics

Data from consecutive STEMI patients who underwent pPCI in a large tertiary hospital were included between January 1, 2005, and January 5, 2007. The pPCI and adjunctive pharmacological treatment was performed according to American College of Cardiology, American Heart Association, and European Society of Cardiology guidelines. In general, patients were eligible for pPCI if they had ischemic chest pain, onset of symptoms less than 12 hours, and at least 1 mm of ST-segment elevation in 2 contiguous leads on the 12-lead electrocardiogram. Patients received aspirin (500 mg), clopidogrel (300 to 600 mg), and unfractionated heparin (5,000 IU). Glycoprotein IIb/IIIa inhibitors were used at the discretion of the operator. If a coronary stent was implanted, clopidogrel was prescribed for a minimum of 1 month to patients after a bare-metal stent placement and for a minimum of 6 months after a drug-eluting stent placement.

In the catheterization laboratory database all data regarding procedural and angiographic characteristics were collected prospectively. The data were entered by interventional cardiologists and trained nurses as part of routine care. The database was consulted for information on baseline patient demographics and angiographic characteristics.

### Ethics Statement

The study was performed under the tenets of the Helsinki declaration, local laws and regulations. The biomarker measurements were performed in de-identified plasma aliquots obtained from the Academic Medical Centre (AMC) Cathlab Biobank, remaining from routine clinical blood sampling. The clinical follow-up was obtained from patient file review. No formal patient informed consent was obtained for the biomarker measurements according to local laws and regulations at the time of the blood drawings. The Cathlab Biobank was approved by the Biobank Instutional Review Board of the AMC – University of Amsterdam. The Dutch Medical Research Involving Human Subjects Act (WMO) did not apply to the clinical follow-up in this study according to the AMC – University of Amsterdam Medical Ethical Review Committee (METC). As a result no formal informed consent was mandatory for the follow-up [13].

### Biomarkers

Blood samples were routinely drawn as part of patient care immediately after insertion of the arterial sheath, before the introduction of the catheter, for assessment of cardiac troponin T (cTnT), C-reactive protein (CRP), glucose, NT-proBNP, and plasma creatinin. Blood samples were centrifuged without undue delay and analyzed. Both cTnT and NT-proBNP were measured using a Hitachi modular E-170 analyzer (Roche Diagnostics GmbH, Mannheim, Germany). CRP was measured with an immunoturbidimetric assay on a Hitachi modular P-800 (Roche Diagnostics GmbH, Mannheim, Germany). Glucose and plasma creatinin were measured with an enzymatic assay on a Hitachi modular P-800 analyzer (Roche Diagnostics GmbH, Mannheim, Germany). Lipid levels were not measured routinely. The eGFR was calculated according to the Cockcroft and Gault formula. Remaining plasma aliquots were coded and stored at −70°C after centrifugation.

### Routine Follow-up

Information on five years vital status obtained from the institutional database, which was synchronized with the Dutch national population registry as part of routine quality control of patient care. The follow-up for all patients was censored at five years of follow-up. Clinical follow-up was available in a subpopulation, including myocardial infarction, cardiac death, stroke and revascularization, up to three years. In short, subjects were included into the subpopulation if a valid activated partial thromboplastin time was available post-procedure, as described earlier [14].

### Study Population and Subpopulation

The complete cohort consists of all consecutive STEMI patients (n = 1340) who underwent pPCI between January 1, 2005, and January 5, 2007. Only the first pPCI was included in the case of a patient with multiple pPCIs within the study period. STEMI patients with cardiogenic shock (n = 85) and patients undergoing rescue PCI after failed thrombolysis (n = 10) were excluded.

In a total of 1012 patients with available plasma aliquots and complete biomarker measurements prior to pPCI Lp-PLA_2_ activity was measured. In the subpopulation a complete 3-year clinical follow-up was available (n = 567).

### Lp-PLA_2_ Activity Measurements

The Lp-PLA_2_ activity levels were measured in de-identified plasma aliquots which were remaining from the routinely taken blood samples at baseline procedure. The samples were stored at –80°C and were thaw in a fridge at 4°C overnight and centrifuged at 2000 g at 18°C for 10 minutes prior to analysis. Lp-PLA_2_ activity levels were measured using the DiaDexus assay (diaDexus Inc., California, USA) on an Architect C8000 (Abbott Laboratories. Illinois, U.S.A.). The detection range of this assay was at least 0.2–450 nmol/min/mL and the inter assay coefficient of variation was 3.6–5.5%. Samples with high serum indices (hemolyses >0.30 and/or icteria >273 and/or lipemia >4.4) were excluded from further analysis (n = 25).

### Endpoints

The main outcome measure for our current analysis was all-cause mortality before the end of follow-up at five years. The secondary outcome measures were the clinical endpoints at three years of follow-up, including 1) a device oriented composite endpoint of target vessel failure including cardiac death, myocardial infarction, and target vessel revascularization 2) a composite patient oriented endpoint of cardiac death and myocardial infarction. Furthermore, individual endpoints including all-cause mortality, cardiac death, myocardial infarction, stroke, and target vessel revascularization were assessed.

### Statistical Analysis

The study population was divided into three equal groups based on the tertiles of Lp-PLA_2_ activity levels. Tertiles were chosen because of its easy interpretation and sufficient group sizes of more than 300 patients, besides comparability with earlier published studies. Normally distributed continuous variables were reported as mean±SD and compared with the one-way ANOVA. Skewed distributed continuous variables were reported as median and interquartile range and compared with the Kruskal-Wallis test. Categorical variables were reported as number and percentage, and compared with the chi-square test.

Clinical endpoints rates were assessed by the Kaplan-Meier method and censored at date of event or last follow-up date. Clinical endpoints were compared across the Lp-PLA_2_ categories using the log-rank test. The prognostic value of the Lp-PLA_2_ was assessed by investigating the relationship between mortality and the Lp-PLA_2_ activity in Cox proportional-hazards analyses using the lowest tertile as the reference. The proportional hazard assumption was verified by visual estimation in a log of survival time graph. An univariate analysis of Lp-PLA_2_ tertiles_,_ and a multivariable analysis including variables associated with mortality was performed. Variables associated with mortality were depicted according the Thrombolysis In Myocardial Infarction (TIMI) risk score including age, body mass index, history of diabetes mellitus, history of hypertension, systolic blood pressure, heart frequency, anterior infarction, and symptom to open vessel time [15]. In addition, a multivariable analysis with the multimarker risk score, including eGFR, glucose and NTproBNP, was performed. Furthermore, the interactions between Lp-PLA_2_ activity levels and significant different baseline variables in mortality were assessed in a Cox-regression model. For the study population and subpopulation versus the excluded patients inverse probability weighting (IPW) was utilized [16]. The propensity score for the IPW was calculated by logistic regression including the significant different variables between the selected populations and the non-selected population (P<0.05). IPW score was entered into the Cox-regression model as an independent variable to assess the possible impact of the selection out of the complete cohort on the prognostic value of Lp-PLA_2_ activity on clinical outcomes.

## Results

### Study Population

**Figure 1:**
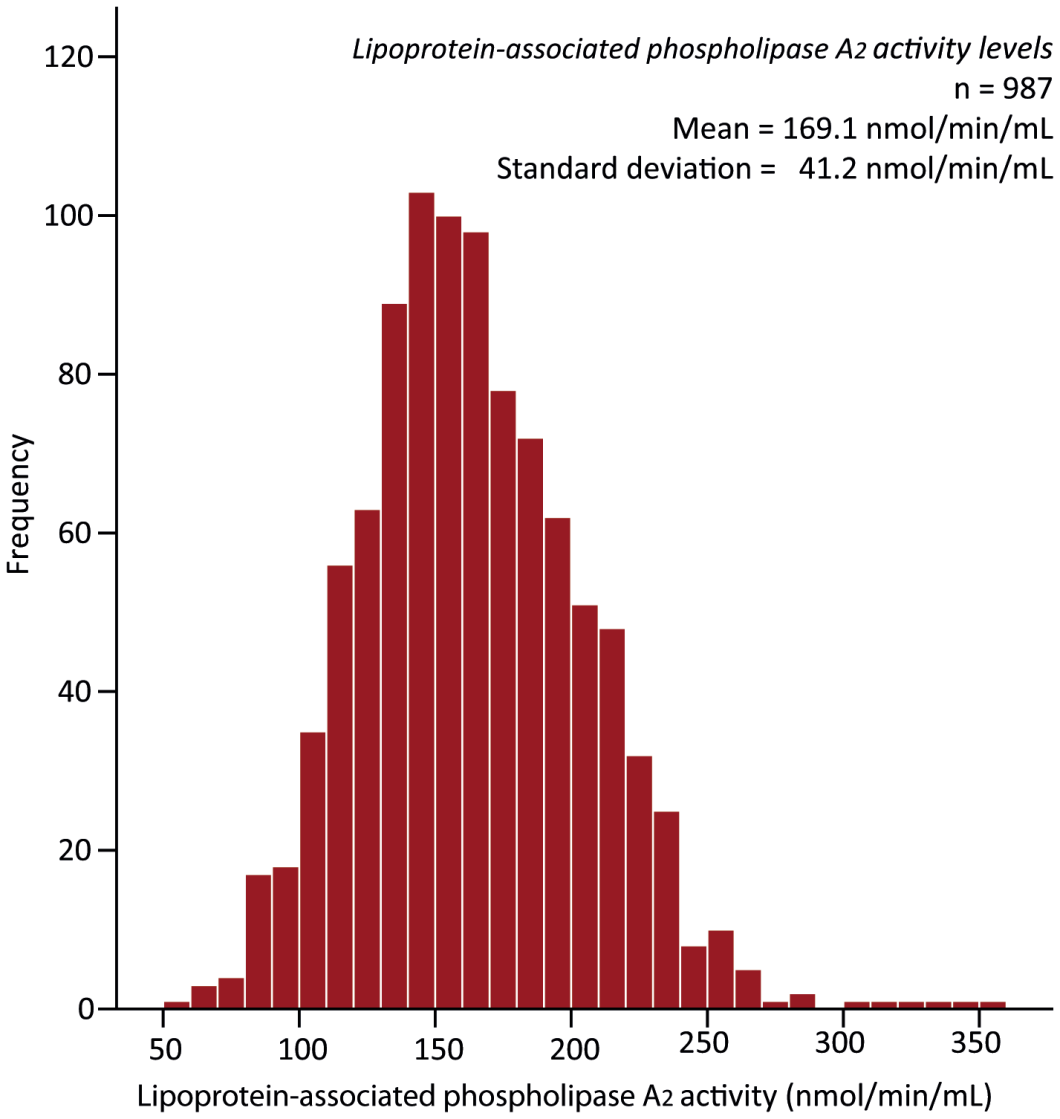
Histogram of Lp-PLA_2_ activity levels in the complete cohort. Histogram showing distribution of measured lipoprotein-associated phospholipase A_2_ activity levels. **nmol/min/mL**, nanomol per minute per milliliter.

In the current analysis 987 patients with valid Lp-PLA_2_ activity measurements were included. The mean (±SD) Lp-PLA_2_ activity was 163.9 nmol/min/mL (±41.3) in this cohort with a near to normal distribution (Figure 1). The patient cohort was divided into tertiles with Lp-PLA_2_ activity cut-offs of <144, 144–179, and >179 nmol/min/mL. In patients with and without statins on admission Lp-PLA_2_ activity was respectively 146.5 (±37.2) nmol/min/mL and 167.5 (±41.2) nmol/min/mL. The mean age was 62 (±13) and 72.6% of the patients were male.

In general it should be noticed that there is a significant difference in the prevalence of several baseline characteristics among the Lp-PLA_2_ activity tertiles, which were all associated with clinical outcomes in a STEMI population (Table 1). Importantly, Lp-PLA_2_ activity was both positive and inverse associated with the baseline variables associated with adverse outcomes.

**Table 1:** Baseline Characteristics.

*Tertiles LpPLA_2_ activity (nmol/min/mL)*	Low (<144)	Intermediate (144–179)	High (>179)	*P value*
	*n* = 324	*n* = 331	*n* = 332	
**Demographics**				
Age (years)	62±13	63±13	61±13	0.07
Sex (male)	195(60.2%)	243(73.4%)	179(84.0%)	<0.001
Body mass index (kg/m^2^)	27.0±4.9	26.6±4.2	26.4±3.4	0.19
**Clinical history**				
Prior myocardial infarction	65(20.1%)	42(12.7%)	23(6.9%)	<0.001
Prior PCI	52(16.0%)	26(7.9%)	16(4.8%)	<0.001
Prior CABG	11(3.4%)	7(2.1%)	5(1.5%)	0.26
**Risk factors**				
Current cigarette smoking	112(34.6%)	146(44.1%)	160(48.2%)	0.001
Hypertension	116(35.8%)	98(29.6%)	88(26.5%)	0.03
Dyslipidemia	94(29.0%)	68(20.5%)	63(19.0%)	<0.01
Diabetes mellitus	66(20.4%)	38(11.5%)	24(7.2%)	<0.001
**Medication**				
Aspirin	290(89.5%)	272(82.2%)	268(80.7%)	<0.01
Statin	84(25.9%)	52(15.7%)	35(10.5%)	<0.001
**TIMI risk score factors**				
Time to treatment* (minutes)	186(133–275)	176(128–266)	188(134–270)	0.81
Anterior infarction	110(34.0%)	137(41.4%)	155(46.7%)	<0.01
Systolic blood pressure (mmHg)	133±28	133±27	133±28	0.96
Heart rate (beats/min)	78±18	76±17	77±18	0.16
**Biomarkers**				
Troponin T (µg/l)	0.04(0.04–0.18)	0.04(0.04–0.21)	0.07(0.04–0.29)	0.03
Glucose (mmol/l)	9.0±3.4	8.8±3.0	8.4±3.0	0.03
NT-proBNP (ng/l)	144(57–499)	160(57–556)	144(53–778)	0.93
eGFR (ml/min)	103±42	103±38	109±41	0.03
CRP (mg/l)	3.1(1.5–8.3)	3.1(1.4–6.8)	3.3(1.3–8.1)	0.26
Multimarker risk score**	12±3	12±3	12±4	0.59
**Angiographic risk factors**	n = 268	n = 285	n = 288	
Thrombus pre-procedure	168(62.7%)	187(65.6%)	172(59.7%)	0.35
TIMI flow pre-procedure	0.80±1.17	0.75±1.15	0.85±1.17	0.61
TIMI flow post-procedure	2.87±0.48	2.87±0.48	2.86±0.48	0.99

Values are number(%), mean (SD) or median (interquartile range).

**CABG**, coronary artery bypass grafting; **CRP**, C-reactive protein; **eGFR**, estimated glomerular filtration rate; **Lp-PLA_2_**, Lipoprotein-associated phospholipase A_2_; **NT-proBNP**, N-terminal pro-brain natriuretic peptide; **PCI**, percutaneous coronary intervention; **TIMI**, Thrombolysis In Myocardial Infarction.

* Time of onset of symptoms to open vessel.

** According to Damman et. al. JACC 2011.

The baseline characteristics for the selected study population showed a significant higher incidence of dyslipidemia and aspirin use, and a significant lower incidence of prior coronary artery bypass grafting (CABG) compared with the patients not selected from the complete cohort (Table 2).

**Table 2:** Baseline characteristics complete cohort, study population and subpopulation.

	Complete cohort	Non-study population *(Lp-PLA_2_ activity unavailable)*		Study population *(Lp-PLA_2_ activity available)*			
			Study vs. non-study population	Subpopulation *(Clinical follow-up)*	Non-subpopulation *(Mortality only)*	Sub vs. non-sub population	
	*n* = 1340	*n* = 354	*P* value	*n* = 567	*n = 420*	*P* value	
**Demographics**							
Age (years)	62±13	62±13	0.62	62±13	62±13	0.46	
Sex (male)	968(72.2%)	252(71.2%)	0.63	400(70.5%)	317(75.5%)	0.09	
Body mass index (kg/m^2^)	26.6±4.2	26.5±4.2	0.72	26.6±4.3	26.7±4.0	0.81	
**Clinical history**							
Prior myocardial infarction	181(13.5%)	51(14.4%)	0.59	69(12.2%)	61(14.5%)	0.28	
Prior PCI	121(9.0%)	27(7.6%)	0.33	45(7.9%)	49(11.7%)	0.048	
Prior CABG	39(2.9%)	16(4.5%)	0.04	11(1.9%)	12(2.9%)	0.35	
**Risk factors**							
Current cigarette smoking	563(42.0%)	145(41.0%)	0.66	248(43.7%)	170(40.5%)	0.31	
Hypertension	409(30.5%)	108(30.5%)	1.00	181(31.9%)	121(28.8%)	0.29	
Dyslipidemia	286(21.3%)	62(17.5%)	0.04	130(22.9%)	95(22.6%)	0.91	
Diabetes mellitus	165(12.3%)	38(10.7%)	0.35	75(13.2%)	53(12.6%)	0.78	
**Medication**							
Aspirin	1107(82.6%)	278(78.5%)	0.02	485(85.5%)	345(82.1%)	0.15	
Statin	228(17.0%)	57(16.1%)	0.62	80(14.1%)	91(21.7%)	<0.01	
**TIMI risk score factors**							
Time to treatment* (minutes)	186(132–267)	191(130–262)	0.99	182(130–269)	188(135–271)	0.52	
Anterior infarction	530(39.6%)	129(36.4%)	0.18	251(44.3%)	151(36.0%)	<0.01	
Systolic blood pressure (mmHg)	132±29	130±31	0.17	133±27	133±29	0.93	
Heart rate (beats/min)	77±18	78±19	0.70	78±18	76±17	0.07	
**Biomarkers**							
Troponin T (µg/l)				0.05(0.04–0.21)	0.04(0.04–0.24)	0.68	
Glucose (mmol/l)				8.9±3.1	8.6±3.2	0.15	
NT-proBNP (ng/l)				149(55–621)	151(58–606)	0.90	
eGFR (ml/min)				104±40	105±40	0.71	
CRP (mg/l)				2.9(1.4–7.0)	3.7(1.5–8.8)	0.02	
Multimarker risk score**				12±3	12±3	0.63	
**Angiographic risk factors**	*n* = 1111	*n* = 270		*n* = 516	*n = *325		
Thrombus pre-procedure	682(61.4%)	154(44.4%)	0.21	197(60.6%)	330(64.0%)	0.18	
TIMI flow pre-procedure	0.8±1.16	0.79±1.17	0.96	0.82±1.19	0.78±1.14	0.63	
TIMI flow post-procedure	2.87±0.48	2.87±0.49	0.73	2.88±0.45	2.86±0.50	0.63	

Values are number(%), mean (SD) or median (interquartile range).

**CABG**, coronary artery bypass grafting; **CRP**, C-reactive protein; **eGFR**, estimated glomerular filtration rate; **Lp-PLA_2_**, Lipoprotein-associated phospholipase A_2_; **NT-proBNP**, N-terminal pro-brain natriuretic peptide; **PCI**, percutaneous coronary intervention; **TIMI**, Thrombolysis In Myocardial Infarction.

* Time of onset of symptomps to open vessel.

** According to Damman et. al. JACC 2011.

### Subpopulation

In the subpopulation, 567 patients with a valid Lp-PLA_2_ activity measurement and a complete clinical follow-up were included. The mean (±SD) Lp-PLA_2_ activity was 162.7±39.2 nmol/min/mL) versus 165.5±43.9 nmol/min/mL in the non-subpopulation (p = 0.29).

Compared with those not selected for the subpopulation, the baseline characteristics in the subpopulation were significantly different for a history of PCI, anterior infarct, for the use of statin and CRP-levels (Table 2).

### Study Population: Association between Lp-PLA_2_ Activity and Mortality

**Figure 2:**
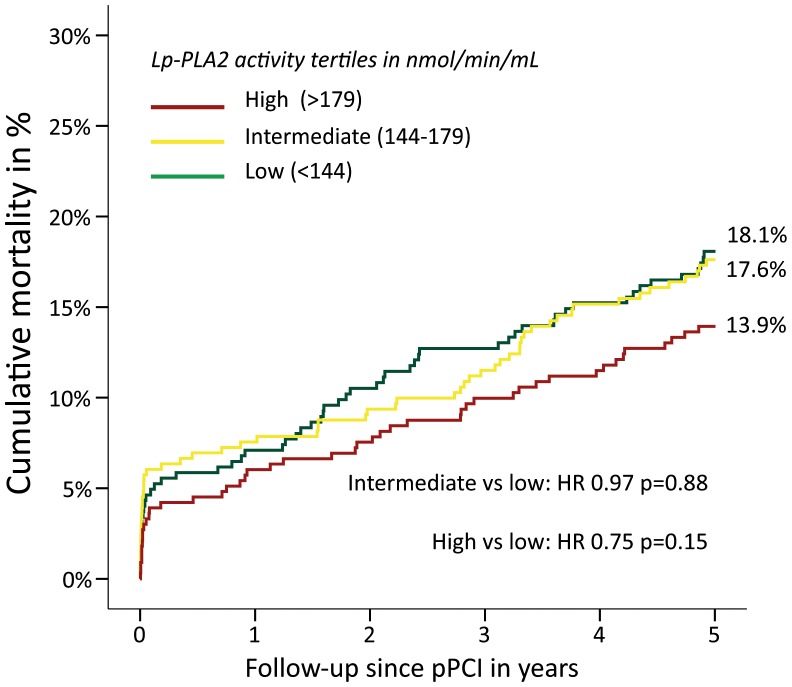
Kaplan-Meier estimates for mortality according to Lp-PLA_2_ activity categories. Kaplan-Meier estimates showing overall mortality from pPCI until the end of follow-up. Non-significantly different hazard ratios shown according to Cox regression estimates. **HR**, Hazard ratio; **Lp-PLA_2_**_,_ Lipoprotein-associated phospholipase A_2_ activity; **nmol/min/mL**, nanomol per minute per milliliter; **pPCI**, Primary percutaneous coronary intervention.

During the complete follow-up 162 deaths occurred. In the univariate analysis no differences were observed when comparing the intermediate with the lowest category (HR 0.97; CI 95% 0.68–1.40; p = 0.88) or the highest with the lowest category (HR 0.75; CI 95% 0.51–1.11; p = 0.15) (Table 3–4, Figure 2). The proportional hazard assumptions were formally violated in the log of survival time graphs, however this was expected because of very parallel and similar curves among the tertiles. Therefore these formal violations do not alter the conclusions of the analyses. Furthermore, in the interaction analyses only heart frequency did have a modest statistical significant interaction with Lp-PLA_2_ categories in mortality (HR1.012, CI95% 1.001–1.023, P = 0.026).

**Table 3:** Unadjusted and adjusted hazard ratios for all-cause mortality at 5-years follow-up for the complete study population with available Lp-PLA_2_ activity.

	All cause mortality		
	HR	(95% CI)	*P* value
**Unadjusted**			
Low Lp-PLA_2_ activity (<144)		Reference	
Medium Lp-PLA_2_ activity (144–179)	0.97	(0.68–1.40)	0.88
High Lp-PLA_2_ activity (>179)	0.75	(0.51–1.11)	0.15
**Adjusted for IPW selected population** *			
Low Lp-PLA_2_ activity (<144)		Reference	
Medium Lp-PLA_2_ activity (144–179)	0.96	(0.67–1.38)	0.82
High Lp-PLA_2_ activity (>179)	0.74	(0.51–1.10)	0.13
IPW selected population	2.28	(0.44–11.8)	0.33
**Adjusted for TIMI risk factors** **			
Low Lp-PLA_2_ activity (<144)		Reference	
Medium Lp-PLA_2_ activity (144–179)	1.07	(0.69–1.66)	0.75
High Lp-PLA_2_ activity (>179)	0.75	(0.46–1.21)	0.23
Age	1.07	(1.06–1.09)	<0.001
Body mass index	1.00	(0.95–1.04)	0.84
History of diabetes mellitus	1.10	(0.68–1.77)	0.70
History of hypertension	1.17	(0.78–1.75)	0.45
Systolic blood pressure at admission	0.99	(0.99–1.00)	0.04
Heart frequency at admission	1.03	(1.02–1.04)	<0.001
Anterior myocardial infarction	0.94	(0.64–1.38)	0.76
Symptom to open vessel time	1.00	(1.00–1.00)	0.04
**Adjusted for mutimarker score**			
Low Lp-PLA_2_ activity (<144)		Reference	
Medium Lp-PLA_2_ activity (144–179)	0.94	(0.65–1.35)	0.72
High Lp-PLA_2_ activity (>179)	0.88	(0.60–1.29)	0.51
Multimarker score	1.42	(1.34–1.50)	<0.001

Values are hazard ratio's (95% confidence interval).

**IPW**, inverse probability weighting; **Lp-PLA_2_**, Lipoprotein-associated phospholipase A_2_; **TIMI**, Thrombolysis In Myocardial Infarction.

*IPW for selection cases with biomarkers available from complete cohort.

**established by Damman et. al. JACC 2011.

**Table 4:** Cumulative event rates for 3 and 5 years follow-up.

*Tertiles Lp-PLA_2_ activity (nmol/min/mL)*	Low (<144)		Intermediate (144–179)		High (>179)			P value*
*5-years follow-up*	n = 324		n = 331		n = 332			
All-cause mortality	58	18.1%	58	17.6%	46	13.9%		0.29
0–30 days	15	4.9%	20	6.1%	13	4.3%		0.42
30 - end of follow up	43	14.1%	38	12.3%	33	10.4%		0.37
*3-years follow-up*	n = 182		n = 203		n = 182			
*Composites*								
Cardiac Death and MI	31	17.4%	41	20.3%	26	14.4%		0.27
Cardiac Death, MI and TVR	36	20.2%	46	22.8%	32	17.8%		0.41
*Individual endpoints*								
All-cause mortality	23	12.7%	30	14.8%	19	10.5%		0.42
Non-cardiac death	10	5.8%	7	3.8%	7	4.1%		0.60
Cardiac death	13	7.3%	23	11.4%	12	6.7%		0.18
Myocardial infarction	23	13.5%	23	12.1%	15	8.6%		0.35
Stroke	8	4.6%	5	2.7%	5	2.9%		0.52
Target vessel revascularization	7	4.1%	7	3.8%	7	4.0%		0.99

Values are *n* (%).

**MI**, Myocardial infarction; **Lp-PLA_2_**, Lipoprotein-associated phospholipase A_2_; **TIMI**, Thrombolysis In Myocardial Infarction; **TVR**, Target vessel revascularization.

*According to log-rank by Kaplan-Meier estimates.

The following variables were included in the regression analysis to calculate the IPW score to correct for the selection of the study population out of the complete cohort; Prior CABG, history of dyslipidemia, and aspirin use. When the IPW score was introduced into the Cox-regression model no substantial changes in hazard ratios of Lp-PLA_2_ activity were observed (intermediate versus low [HR 0.96; CI 95% 0.67–1.38; p = 0.82] and high versus low [HR 0.74; CI 95% 0.51–1.10; p = 0.33]) with an non-significant association for the IPW score for the selection from the complete cohort (HR 2.28; CI 95% 0.44–11.8; p = 0.33).

**Figure 3:**
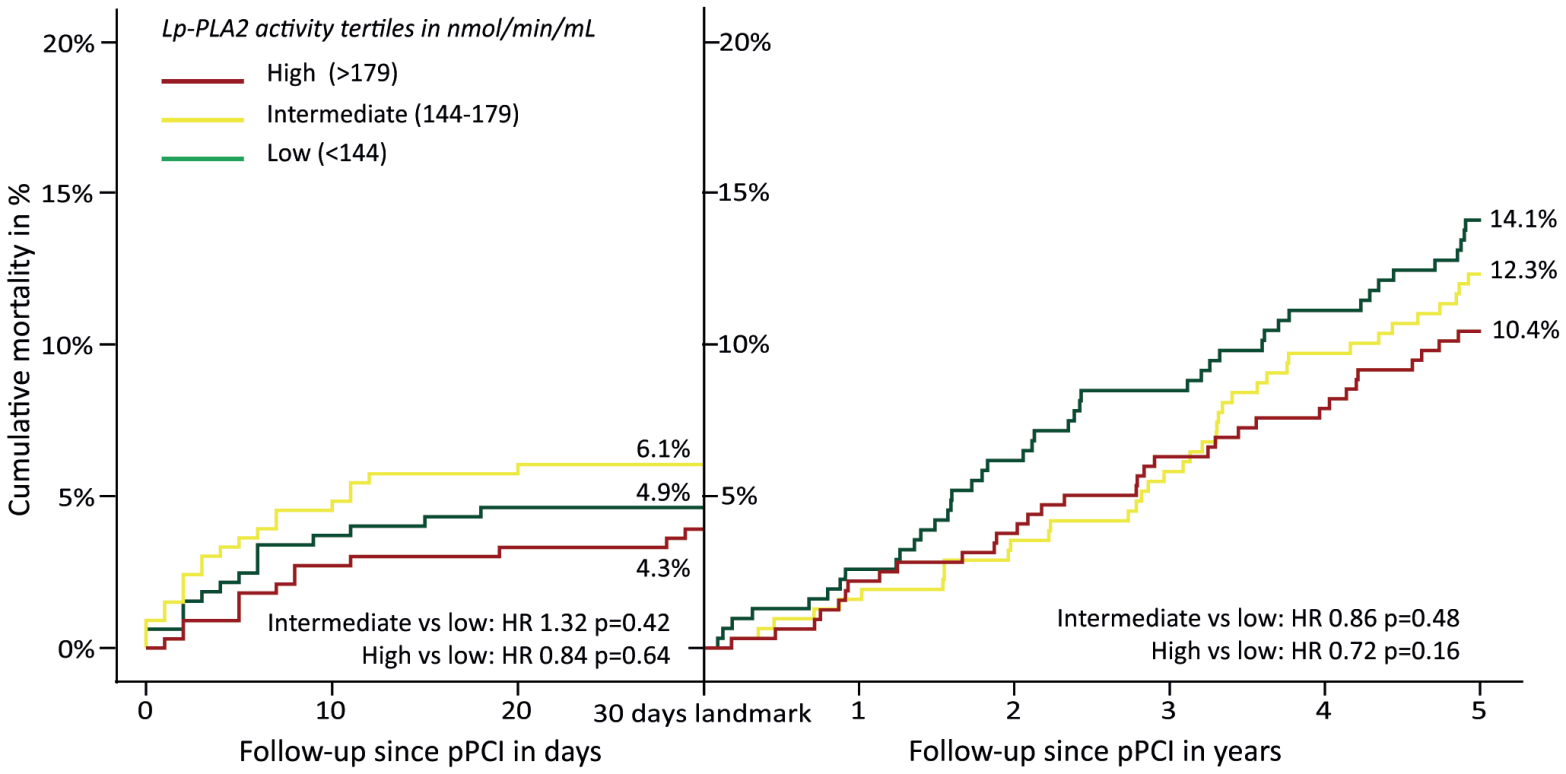
Landmark Kaplan-Meier estimates for mortality according to Lp-PLA_2_ activity categories. Kaplan-Meier estimates showing overall mortality with a landmark at thirty days follow-up. Non-significantly different hazard ratios shown for both follow-up periods according to Cox regression estimates. **HR**, Hazard ratio; **Lp-PLA_2_**, Lipoprotein-associated phospholipase A_2_ activity; **nmol/min/mL**, nanomol per minute per milliliter; **pPCI**, Primary percutaneous coronary intervention.

When a landmark analysis was performed, comparable mortality rates were observed at 30 days (intermediate versus low [HR 1.32; CI 95% 0.67–2.57; p = 0.42] and high versus low [HR 0.84; CI 95% 0.40–1.76; p = 0.64]) (Figure 3). Furthermore, at follow-up from 30 days until the end of follow-up non-significant differences between the groups were observed (intermediate versus low [HR 0.85; CI 95% 0.55–1.32; p = 0.48] and high versus low [HR 0.72; CI 95% 0.46–1.14; p = 0.16]) (Figure 3).

When established risk factors comprised in the TIMI risk score were added in a multivariate analysis, no differences were observed between the intermediate versus the low category (HR 1.07; CI 95% 0.69–1.67; p = 0.76) or the high versus the low category (HR 0.74; CI 95% 0.45–1.24; p = 0.25) (Table 3). Lastly, when adjusted for the multimarker risk score which was a significant predictor for mortality (HR 1.42; CI 95% 1.27–1.39; p<0.001), no differences were observed in the intermediate versus the low category (HR 0.94: CI 95% 0.65–1.35; p = 0.72) or the high versus the low category (HR 0.88; CI 95% 0.60–1.29; p = 0.51) (Table 3).

### Subpopulation: Association between Lp-PLA_2_ Activity and Clinical Endpoints

In the subpopulation no significant differences were found among the tertiles in the occurrence of the composite endpoint of cardiac death and MI (low 17.4%, intermediate 20.3%, high 14.4%, p = 0.27). The composite endpoint of target vessel failure was not significantly different among the groups (low 20.2%, intermediate 22.8%, high 17.8%, p = 0.41). In addition, the individual endpoints were not significantly different among the tertiles (Table 4).

The following variables were included in the regression analyses to calculate the IPW to correct for the selection of the subpopulation out of the study population; Prior PCI, statin use, anterior infarction, and CRP level. The IPW score for selection from the complete cohort was entered into the Cox-regression models without any significant changes in hazard ratios for all events among the tertiles, and no significant association of the IPW score with the endpoints.

## Discussion

The current analyses provide no evidence for an association of Lp-PLA_2_ activity, measured at admission before pPCI for STEMI, with long-term all-cause mortality. In a subgroup, no associations between Lp-PLA_2_ activity and clinical endpoints at 3 years, including cardiac death, MI, stroke, or revascularization, were observed. The results were consistent when corrected for known predictors of mortality in STEMI patients.

### Cardiovascular Disease and Lp-PLA_2_

Prospective epidemiological studies have investigated the association between circulating Lp-PLA_2_ and the subsequent risk of vascular disease outcomes. A large meta-analysis did combine the individual records of 32 available prospective studies [4]. In short, Lp-PLA_2_ activity and mass were associated with each other and with the risk of coronary heart disease, similar in magnitude to that with non-HDL cholesterol or systolic blood pressure in this population. These observations were true for the complete group of healthy individuals, and for patients with a history of stable vascular disease. However, in the subgroup of patients with acute ischemic events there was no association between baseline value of Lp-PLA_2_ and vascular disease outcomes. The results of this meta-analysis are in accordance with the results of the current study, although the patient groups differ significantly.

### Acute Ischemic Disease and Lp-PLA_2_

Several studies have been published with regard to Lp-PLA_2_ mass and activity levels in ACS (Table 5). One of the first studies to be published was the Olmsted County registry which prospectively identified and followed patients (n = 271) who experienced a MI between 2003 en 2005 [17]. Lp-PLA_2_ mass was measured in frozen samples early after MI. After adjustment for age and sex, the hazard ratios for death in the middle an upper Lp-PLA_2_ tertiles were 2.20 (95% CI; 0.88–5.54) and 4.93 (95% CI; 2.10–11.60), compared with the lowest tertile (*P*_trend_ <0.001). In this study, Lp-PLA_2_ mass levels measured shortly after MI were strongly and independently associated with one year mortality and provided incremental value in risk discrimination over traditional predictors. To best of our knowledge this is the only study to report a positive association between Lp-PLA_2_ levels in the direct timeframe of acute ischemic disease and mortality.

**Table 5:** Previous studies in acute coronary syndrome.

Authors	Year ofpublication	Journal	Number ofpatients	Population	Samplingmoment	Follow-up	Hazard ratio/event rate mortality			
							Lp-PLA_2_ Mass		Lp-PLA_2_ activity	
Gerber et. al.	2006	Arterioscler ThrombVasc Biol	271	MI	Baseline	1 year	Lowest vs. Middle andHigh tertiles	1.92 (0.77–4.82), 3.48 (1.49–8.14)*P*trend = 0.003	*na*	*na*
O'Donoghue et. al.	2006	Circulation	3648	ACS	Enrollmentpost-PCI	Mean 24 months	*na*	*na*	Quintiles, adjustedhigh vs low	0.65 (0.33–1.28, p = 0.21)
Oldgren et. al.	2007	Eur Heart J.	2266	ACS	Post-randomization	1 year	Tertiles, high vs low	1.4 (0.77–2.5, p = 0.3)	*na*	*na*
Ryu et. al.	2012	Circulation	2587	ACS	mean 63hours	16 weeks	Doubling of level	1.07 (0.72–1.58, p = 0.17)	Doubling of level	0.91 (0.52–1.59, p = 0.73)
Stankovic et. al.	2012	Clin. Lab	100	ACS	Admission	30 days	Low vs. High (463 ng/mL)	0% vs. 18.6% (p<0.001)	*na*	*na*

Three large studies reported non-significant associations between baseline Lp-PLA_2_ levels in ACS and outcomes. The first was the PROVE IT-TIMI 22 (PRavastatin Or atorVastatin Evaluation and Infarction Therapy – Thrombolysis) trial, in which Lp-PLA_2_ activity levels were measured at baseline (n = 3648) and 30 days (n = 3265) in patients randomized to atorvastatin 80 mg/d or pravastatin 40 mg/d after ACS [18]. The baseline Lp-PLA_2_ levels measured after the initial ACS event did not predict for the risk of recurrent cardiovascular events (death, myocardial infarction, unstable angina, revascularization, or stroke) at a follow-up from 18 to 36 months (mean 24 months). Though, Lp-PLA_2_ activity levels at 30 days were lowered with high-dose statin therapy and Lp-PLA_2_ activity levels were associated with an increased risk of cardiovascular events.

Second, in non-ST-segment elevation ACS patients in FRISC II (Fast Revascularisation during InStability in Coronary artery disease, n = 1326) and GUSTO IV (Global Utilization of STrategies to Open occluded arteries, n = 904) trials Lp-PLA_2_ mass levels were measured at randomization [19]. In the pooled dataset, and in both studies separately, no significant association was found between Lp-PLA_2_ levels and 1 year mortality (HR 1.4 CI 95%: 0.77–2.5, p = 0.3 for the natural logarithm of Lp-PLA_2_).

The results of the MIRACL (Myocardial Ischemia Reduction with Acute Cholesterol Lowering) trial on the assessed biomarkers were reported [20]. In this trial Lp-PLA_2_ mass and activity were measured at baseline (24 hours to 96 hours after hospital admission) and after 16 weeks of treatment with atorvastatin 80 mg/day or placebo in patients with ACS (n = 2587). Baseline levels of Lp-PLA_2_ were not associated with the primary efficacy endpoint of death, myocardial infarction or unstable angina at 16 weeks.

Lastly, a study in STEMI patients measured Lp-PLA_2_ mass in 100 patients at admission [21]. Low and high Lp-PLA_2_ mass levels (low: <463 ng/mL, n = 67; and high: ≥463 ng/mL, n = 33) were associated with 30-day outcomes. PCI was performed within 6 hours after onset of symptoms. Both mortality and MACE rates were significantly lower in the low Lp-PLA_2_ group versus the high Lp-PLA_2_ group. Moreover, multiple logistic regression analysis identified the plasma Lp-PLA_2_ level as an independent predictor of MACE (OR 1.011; 95%CI 1.001–1.013; p = 0.037). Although this analysis shows an significant prognostic value of a high Lp-PLA_2_ at baseline, several limitations need to be mentioned; Lp-PLA_2_ mass is only measured in a small group of patients in a very low volume center with a possible long delay to PCI, up-to 6 hour, with a very short follow-up. Although contradicting, the observations in our analyses are strengthened by the long-term follow-up in a high volume contemporary practice with a very short time from onset to measurement in a very large population. An alternative explanation could be the absence of an elevation of Lp-PLA_2_ in the early acute phase, in which our measurements were performed. In the above study a longer time to treatment could have induced a possible selection bias, with patients with a long time-to-PCI having both a worse prognosis and a higher Lp-PLA_2._

The current study adds to our knowledge being the first to report on the prognostic value of admission Lp-PLA_2_ on long-term clinical endpoints in patients presenting with STEMI treated with pPCI. In addition, this analysis reflects the prognostic value of Lp-PLA_2_ activity in contemporary practice. Furthermore, the prospectively collected samples were obtained before pPCI in consecutive patients without other study related interventions, as was apparent in several of the earlier studies [18]–[20]. The results of the current study are generally in line with previous results in above mentioned study populations. One of the possible explanations for the lack of predictive value could be found in the absence of an elevation of Lp-PLA_2_ in the early acute phase. This argument is supported by the lack of association in our analyses between the onset of symptoms to open vessel time and Lp-PLA_2_ levels. In contrast, it could also be postulated that the Lp-PLA_2_ activity raise in the acute phase dilutes the prognostic effect. As a result, prognostic value of activity levels could than only be observed at follow-up, such as for example in PROVE IT-TIMI 22 [18].

### Biological Role of Lp-PLA_2_

Studies on the biological role of Lp-PLA_2_ are contradictory, showing both anti-atherogenic and pro-atherogenic functions, and not completely clarified yet. The anti-atherogenic role of Lp-PLA_2_ was indicated by evidence showing that it plays a role in the enzymatic catabolism of biologically active oxidized phospholipids (oxPLs) in LDL and degradation of platelet activating factor [3]. However, more recent evidence suggests an anti-inflammatory role for oxPLs and as a result Lp-PLA_2_ might promote inflammation by the hydrolysis of oxPLs [1], [22]. In a patient setting Lp-PLA_2_ deficiency has been described in the Japanese population, being associated with increased risk of developing atherosclerosis and its clinical manifestations including myocardial infarction and stroke [23], [24]. These results support the concept of a protective effect of Lp-PLA_2_.

### Clinical Implications

The current analysis implicates that Lp-PLA_2_ has no role in prognostication in the acute phase of an ACS. Notably, several baseline characteristics associated with long-term outcomes are significantly different among the tertiles. However, these differences did not influence the prognosis of the groups, probably because there was a mix of variables with positive associations and with negative associations with regard to clinical outcomes [17], [18]. In contrast, it has a prognostic role in stable vascular disease or stabilized ACS, and might therefore be a potential therapeutic or preventive target. First promising results have been published on inhibitors of forms of phospholipase A_2_ [25], [26]. Currently large multicenter trials are undertaken with these new drug compounds to prove clinical benefits of phospholipase A_2_ inhibitors in large scale populations [27]–[29]. Notably, the phase III STABILITY (STabilisation of Atherosclerotic plaque By Initiation of darapLadIb TherapY) randomized controlled trial on the efficacy of a Lp-PLA_2_ inhibitor has recently been terminated on the basis of no likelihood of efficacy at the time of a prespecified interim analysis.

### Limitations

Several limitations of the current study deserve to be mentioned. First, the Lp-PLA_2_ levels have been assessed in frozen serum samples retrospectively and routine lipids levels are not available. However, all baseline data and samples were collected systematically and prospectively. Second, there is a potential selection bias because of the exclusion of patients with missing or incomplete biomarkers. However, although some significant differences among baseline variables were observed, the IPW corrected analyses did not change the outcomes. The near normal distribution of Lp-PLA_2_ activity is another argument to expect this cohort to be a representative sample. Third, the proportional hazard assumption is violated, limiting the interpretation of the Cox-models and hazard ratios. However, the violation is mainly caused by overlapping and parallel survival curves at each time point, as a result small changes by change cause violations of the assumption without any consequences for the clinical interpretation of the results or conclusions of the analyses. Fourth, only Lp-PLA_2_ activity levels, and no Lp-PLA_2_ mass, have been evaluated. Nonetheless, a previous study showed a good correlation between Lp-PLA_2_ mass and Lp-PLA_2_ activity with similar assays [20]. Fifth, information on vital status in the study population was obtained from the Dutch national population registry, wherein information on the cause of death is not available. Finally, only from a subpopulation follow-up including additional clinical endpoints was available. However, overall baseline characteristics were comparable, in the subpopulation no differences in clinical endpoints were found among the tertiles, and there was a similar distribution of mortality in the subpopulation compared with mortality at five years follow-up in the complete cohort. Furthermore, the analyses corrected for the IPW score for selection from the total cohort show similar results.

## Conclusion

The levels of Lp-PLA_2_ activity in STEMI patients before pPCI are associated, both positive and inversely, with differences in baseline patient characteristics which have been associated with cardiovascular mortality. However, the Lp-PLA_2_ activity levels obtained in the acute phase of STEMI are not associated with short- or long-term clinical endpoints, including mortality, myocardial infarction, stroke or target vessel revascularization. Lp-PLA_2_ as an independent and clinically useful biomarker in the risk stratification of STEMI patients still remains to be proven.

## Supporting Information

Checklist S1 **STROBE Checklist.**
(DOC)
